# City puzzles: Does urban land scape affect genetic population structure in *Aedes aegypti*?

**DOI:** 10.1371/journal.pntd.0010549

**Published:** 2022-07-06

**Authors:** Lucía Maffey, Viviana Confalonieri, Esteban Hasson, Nicolás Schweigmann

**Affiliations:** 1 Grupo de Estudio de Mosquitos, Departamento de Ecología, Genética y Evolución (DEGE-UBA)/Instituto de Ecología, Genética y Evolución de Buenos Aires (IEGEBA-CONICET), Buenos Aires, Argentina; 2 Grupo de Investigación en Filogenias y Filogeografía, Departamento de Ecología, Genética y Evolución (DEGE-UBA)/Instituto de Ecología, Genética y Evolución de Buenos Aires (IEGEBA-CONICET), Buenos Aires, Argentina; 3 Laboratorio de Evolución, Departamento de Ecología, Genética y Evolución (DEGE-UBA)/Instituto de Ecología, Genética y Evolución de Buenos Aires (IEGEBA-CONICET), Buenos Aires, Argentina; Kenya Agricultural and Livestock Research Organization, KENYA

## Abstract

Cities usually offer a suitable environment for the dengue vector *Aedes aegypti*, providing oviposition sites, accessibility to human hosts and nectar meals. However, large urban centres are highly heterogeneous environments, forming a patched landscape that could affect *Ae*. *aegypti* population dynamics and dispersal. Here, we performed a genome-wide analysis using Rad-seq data from 99 *Ae*. *aegypti* specimens collected in three areas within Buenos Aires city with varying levels of urbanization/land use: highly urbanized Area 1, intermediate Area 2 and poorly urbanized Area 3. We found an inverse association between urbanization levels and spatial genetic structure. Populations from highly urbanized Area 1 did not present genetic structure whereas two and three clusters were detected in Areas 2 and 3, respectively. In the case of Area 3, initial analyses showed separation in clusters was mostly due to elevated consanguinity within sites although three clusters were still detected after closely related individuals were discarded. Mosquitoes around each site displayed a high degree of isolation, evidencing a close dependence between the vector and human dwellings. Interestingly, specimens from distant boroughs (within the limits of the city) and the city’s outskirts formed a single cluster with inner city sites (Area 1), highlighting the role of passive transport in shaping population structure. Genetic distances were poorly correlated with geographic distances in Buenos Aires, suggesting a stronger influence of passive than active dispersal on population structure. Only Area 2 displayed a significant isolation-by-distance pattern (p = 0.046), with males dispersing more than females (p = 0.004 and p = 0.016, respectively). Kinship analyses allowed us to detect full-siblings located 1.5 km apart in Area 1, which could be due to an extreme event of active female dispersal. Effective population size was higher in Area 2 confirming that cemeteries represent highly favourable environments for *Ae*. *aegypti* and need to be specifically targeted. Our results suggest that control programs should take into account urban landscape heterogeneity in order to improve vector control.

## Author summary

Arboviral vector *Aedes aegypti* usually thrives in urban areas due to its close dependence of human dwellings on breeding sites and meals. However, urban landscapes are heterogeneous and present varying levels of urbanization and land use, affecting *Ae*. *aegypti* spatial structure and dispersal. We used nuclear Single Nucleotide Polymorphisms to analyze 99 *Ae*. *aegypti* specimens from three areas within the city of Buenos Aires: highly urbanized Area 1, intermediate urbanized Area 2 and poorly urbanized Area 3. We found an inverse association between urbanization levels and spatial genetic structure: mosquitoes from Area 1 did not present genetic structure. On the other hand, mosquitoes from Area 2 and 3 displayed 2 and 3 clusters, respectively. Human-mediated transport plays an important role in highly urbanized areas, connecting mosquito populations from distant locations. Potential active dispersal was detected in Area 1 where we found pairs of full-siblings located 1500m apart. In less urbanized areas, the distribution of breeding sites also affected spatial genetic structure. In Area 3, spatial structure was mostly due to consanguinity, but it also reflected the effect of urban landscape on *Ae*. *aegypti* population dynamics. Area 2 presented the highest effective population size. This could be partially explained by the advantageous conditions that cemeteries offer to vector populations which can also spill over surrounding neighbourhoods. Our results suggest that vector programs should consider the heterogeneity of urban landscapes to improve the effectiveness of control measures.

## Introduction

During the last fifty years the world has witnessed an unprecedented emergence/re-emergence of arboviral diseases [1]. Highly anthropophilic *Aedes (Stegomyia) aegypti* constitutes the primary vector of these pathogens in urban environments. Ancestral populations of *Ae*. *aegpyti* (currently known by the sub-species denomination of *formosus*) are found in African forests, breeding in tree-holes and feeding on non-human hosts [2]. More recently, *Ae*. *aegpyti* was the first African species within the genus *Aedes* that evolved to exploit the novel niche associated with human villages, using artificial containers as oviposition sites and developing a distinct predilection for human-blood meals [3]. Currently, the most accepted hypothesis is that the domestication of *Ae*. *aegypti* occurred in West Africa prior to the posterior migration of the species first to America and then Asia. Today *Ae*. *aegypti* has efficiently spread to tropical, subtropical and temperate domestic habitats worldwide [4]. Arboviral expansion has been associated with the spread of *Ae*. *aegypti* and its ability to colonize new areas [5]. Effectively, *Ae*. *aegypti’*s distribution fluctuates and grows mainly due to human-mediated migration and increasing temperatures caused by global warming [6].

In urban areas, the population dynamics of *Ae*. *aegypti* are driven by climate conditions and environmental features [7] which are highly heterogeneous. Different degrees of urbanization, vegetation coverage and land use coexist within large cities, forming a patched landscape that conditions mosquito abundance, dispersal and, consequently, disease transmission at a microgeographic scale [8,9]. Urban landscapes determine the availability and distribution of breeding sites, quality of larval habitats and exposure of human hosts [9–11]. The prevalence of impervious surfaces is also directly related to the urban heat island effect which affects microclimate temperature in mosquito breeding sites [9]. Dispersal of *Ae*. *aegypti* throughout urban areas also depends on different physical and social features that either obstruct or facilitate the movement of mosquitoes [7]. Given that *Ae*. *aegypti* active dispersal is generally limited, human connectivity within and between urban areas plays a crucial role in shaping population dynamics through passive transport of mosquito specimens [12].

With the exception of yellow fever, no safe vaccine or specific treatment is available for arboviral diseases. Therefore, prevention depends almost exclusively on vector control. Genetic characterization of local populations is crucial to anticipate disease expansion and to improve current vector control strategies [6]. Spatial genetic structure is determined by the interplay between active/passive vector dispersal, connectivity/isolation between populations, migration and introduction of novel alleles, bottlenecks and past extinction events and temporal overwintering dynamics [13]. Understanding the outcome of these interactions is paramount for implementing a successful control program against *Ae*. *aegypti* at the local level. Previous studies analyzed *Ae*. *aegypti* population genetic structure within cities using nuclear markers such as microsatellites [14–21] or SNPs [20,22–25]. While most of these works did not focus on the effect of urban heterogeneity on vector’s genomics, those that did showed a clear effect of urban landscape on genetic structure and vector dispersal [18,24].

Buenos Aires represents the main urban conglomerate in Argentina and one of the largest metropolises in the world (United Nations, 2016). The urban matrix is composed of paved streets and buildings with patches of green spaces such as parks, squares, reserves and one university campus. The city’s cemeteries are located within highly urbanized neighborhoods as a result of rapid and unplanned urbanization processes [26]. Currently, Buenos Aires harbors a well-established and growing population of *Ae*. *aegypti*. After continental vector control programs were discontinued in South America, *Ae*. *aegypti* reinvaded its previous range and even expanded to cooler areas [27]. In Argentina, it was first detected in the Northeastern province of Formosa in 1986. Since then, it colonized almost all northern and central provinces of the country [28]. *Ae*. *aegypti* first reached Buenos Aires in 1995 [29] and only one year later it had expanded throughout the city [30]. As in most temperate areas, reproduction and development are conditioned by temperature and only occur during the warmer months, from late September to late June [31]. During winter, it is assumed that the population remains mainly as eggs; only a few larvae have been reported during this season [32]. Previous studies in this area addressed vector abundance [33,34] and the relationship between infestation levels and environmental features within urban sites [26]. However, none of them took into account *Ae*. *aegypti* population genetic structure or potential dispersal ranges.

Here, we sought to investigate the genetic structure of *Ae*. *aegypti* in different urbanized areas of Buenos Aries to understand the role of urbanization in the dispersal and genetic structure of the vector. To achieve this goal, we used high-throughput nuclear sequence data to evaluate population genetic structure on the basis of a genome-wide analysis in three areas within the city of Buenos Aires with varying environmental and social conditions. We hypothesized that landscape heterogeneity can affect the spatial genetic structure and dispersal range of *Ae*. *aegypti* in urban areas, since anthropic modifications to the physical environment usually modulate population structure [18] either impeding or facilitating vector dispersal and gene flow. Understanding the dispersal behavior of *Ae*. *aegypti* is key to improve any control strategy. Fine-scale genetic structure can provide critical information about the potential dissemination of alleles associated to insecticide resistance, particularly those of the *kdr* gene [35,36], and vector competence for different arboviruses [7]. It also conditions the potential success of vector control programs based on the release of *Wolbachia*-carrying or genetically modified mosquitoesto limit arboviral transmission [37]. Tracking mosquito dispersal and its underlying mechanisms can also help improve traditional methodologies based on breeding-site elimination by determining passive transport routes and active dispersal range in different areas within a city. Finally, additional samples from presumptively older *Ae aegypti* populations from Northeastern and Northwestern Argentina [38] were included to address vector movement at a countrywide level.

## Materials and methods

### Study areas

**Fig 1:**
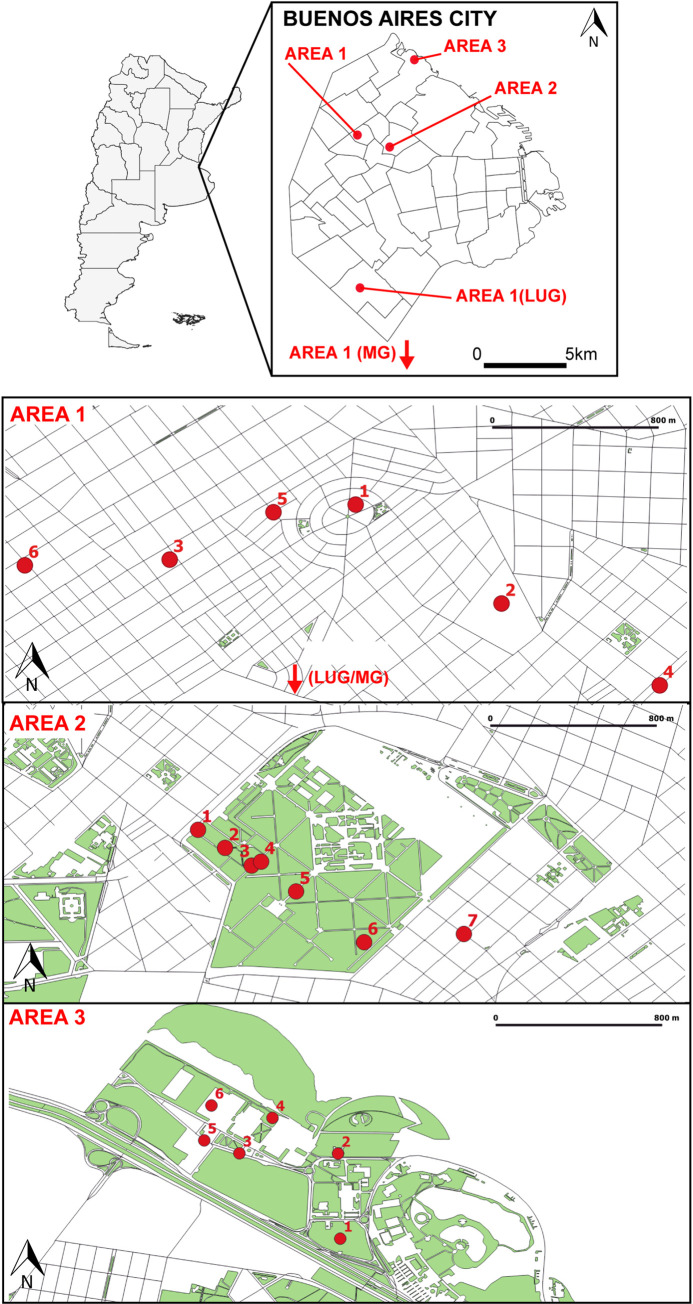
Collection sites for *Ae*. *aegypti* in different areas within Buenos Aires city. Sampling sites in each area are depicted with red dots. In Area 1, collection sites located in Monte Grande (MG), and Lugano (LUG) are not directly depicted in the map for scale purposes. Maps were created using Qgis and layer files were all retrieved from public domain source available at: https://data.buenosaires.gob.ar/dataset/.

We selected three environmentally heterogeneous areas (Area 1, Area 2 and Area 3) within Buenos Aires city (Fig 1) (S1 Table) which present different levels of urbanization and vegetation cover and variable availability of human hosts and potential breeding sites. Area 1is located in the highly urbanized borough of Parque Chas (Fig 1A), situated in the geographic centre of the city. This part of the city combines one or two story houses usually with a backyard and higher buildings, mainly on avenues and two-way streets. Vegetation cover is low (less than 5%) [39] and there is high availability of human hosts and potential breeding sites for the vector. Here, mosquitoes were sampled within a 1 km^2^ area comprising 4 four sampling points during late 2017 season (S2017) and four sampling points during early 2018 season (S2018). During S2017, we also obtained samples in two other highly urbanized areas, one located in the distant borough of Villa Lugano (LUG) and another situated in the outskirt town of Monte Grande (MG) 10 and 30 km away from Parque Chas, respectively. We included samples from these sites together with Area 1 samples in genetic structure analyses to obtain an overview about the relationship among *Ae*. *aegypti* populations sampled in highly urbanized neighbourhoods from downtown Buenos Aires and from more distant boroughs or the city’s outskirts.

Area 2 encompassed two cemeteries, Cementerio de Chacarita and Cementerio Británico and one sampling point in the surrounding borough (Fig 1B). Chacarita is the largest cemetery in the city (0.72 km^2^) administered by municipal authorities, whereas Británico is a private cemetery that covers a much smaller area (0.05 km^2^). Both cemeteries are contiguous and are located in a crowded neighbourhood with high buildings and intense commercial activity. Chacarita cemetery is surrounded by walls 5m high and shows various environments. In this case, all sampling points were located within an open field area with grass graves and scarce trees. The average vegetation cover for the whole cemetery is around 26% [26]. Británico cemetery is also surrounded by 5 m high walls and presents higher vegetation cover of around 82% [40]. Both cemeteries are separated by an8 m high wall. The only direct communication between both areas are five semicircular holes of about 0.8 m^2^at ground level located 20 meters apart designed for water draining. Both sampling areas show an intermediate degree of urbanization with scarce buildings on the premises but surrounded by densely populated neighbourhoods. In both cases, cemetery staff provides containers (plastic or metal cones) to use as flower vases although visitors also bring their own containers (mostly ceramic vases or glass flasks). Previous studies detected high abundance of water-holding containers which provide continuous availability of breeding sites for *Ae*. *aegypti* in both areas [26,33,41]. Human hosts are available during daytime although there are no permanent residents in the area.

Area 3 comprised Ciudad Universitaria (CU), a 0.4km^2^ campus that belongs to University of Buenos Aires, situated close to the borough of Núñez (Fig 1C). CU is situated on the banks of Río de la Plata on the eastside and is bordered on the west by two extended highways, which makes it a highly isolated area within a densely populated part of the city. CU includes several buildings with extended parks among them and a natural reserve area. Overall, it presents a low level of urbanization with around 89% of vegetation coverage. Thousands of students and faculty members circulate daily through the premises although this circulation is mainly restricted to specific buildings and little movement among buildings. Regarding permanent residents, during the time of our study only one family lived in site 1whereas a little squatter settlement was located behind site 6. Potential breeding sites for mosquitoes are water-holding containers located mostly around buildings. Therefore, availability of potential oviposition sites for *Ae*. *aegypti* within this area is discontinuous and associated to building patches.

To determine countrywide genetic structure and to infer connectivity between *Ae*. *aegypti* populations in Argentina, we performed a macrogeographic study that included all samples collected in Buenos Aires and samples from Northeastern and Northwestern Argentina. These two regions are considered the main sources of *Ae*. *aegypti* reintroduction in the country from Brazil and Bolivia, respectively, after eradication campaigns were discontinued in the 1970s [42].

### Mosquito sampling and rearing

Samples from Area 1 (N = 29), Area 2 (N = 35) and Area 3 (N = 19) were collected between April and May 2018. To evaluate inter-seasonal variation in the highly urbanized area, a second batch of specimens was collected in December 2018 from four sampling sites in Area 1 (N = 16). Samples from Area 1 and all samples from Area 3 were collected using ovitraps, whereas samples from Area 2 were obtained as larvae from flower vases with the exception of site 7 where specimens were obtained from ovitraps placed on sidewalks. Ovitraps consisted of glass containers partially filled with dechlorinated tap water and a wooden paddle (commercially available as tongue depressor) attached to the inner wall by a clip aimed as oviposition substrate.

Eggs were recovered from ovitraps and transferred to plastic cups containing an aqueous solution of commercial powdered baker’s yeast (10%) to stimulate hatching. All larvae were placed in plastic containers with dechlorinated tap water and fed *ad libitum* with more powdered yeast. Once pupated, larvae were transferred to individual glass flasks until adult emergence. Ninety-nine specimens were selected for genomic DNA extraction using Qiagen QIAamp genomic DNA kit with an RNAse treatment step. No more than three adult specimens for each sensor/container were selected to avoid excessive sampling of closely related individuals.

### ddRADseq library preparation and SNP typing

ddRADseq library preparation was conducted following the guidelines outlined in Peterson *et al* [43] with minor modifications. Genomic DNA (100 ng) was digested with restriction enzymes EcoRI and Pstl. DNA fragments were purified using AMPureXP beads (Agencourt) and then ligated to barcode adapters. Targeted fragment distribution was collected in a BluePippin instrument (Sage Science Inc.). Libraries were examined with Qubit 2.0 Fluorometer (Invitrogen, Carlsbad, CA) and Bioanalyzer DNA assays (Agilent technologies, Santa Clara, CA) and processed with Illumina cBot for cluster generation on the flow cell, following manufacturer’s instructions. Finally, libraries were sequenced inanIlluminaHiSeq2500 using 125 bp paired-end chemistry.

Raw fastq sequences were processed using a customized pipeline that retained reads with phred scores ≥ 10 and trimmed to 110 bp. High-quality reads were aligned to the *Ae*. *aegypti* reference nuclear genome (AaegL5.0) [44] using the short read aligner software BWA v0.7.17 (BWA-MEM algorithm) [45] with default parameters. Uniquely aligned reads were analyzed with STACKS v2.1 [46] which we used to call genotypes on RAD stacks with a minimum read depth of three. We ran the *populations* program included in Stacks with–r = 0.75 to retain loci that were present in ≥ 75% of the samples and export the resulting files. Subsequent filtering aimed to retain individuals with <25% missing data and minor allele frequency >0.1 was performed with plink [47]. We employed the same software tofurther filter this dataset by eliminating linked loci using the—indep parameter (SNP window size = 500, window shift size = 50, variance inflation factor = 2) and then evaluated the dataset with the—r2 command as previously described [48]. The final Rad-seq data set included 99 individuals and4836 unlinked and informative SNPs for further analyses. Because highly related individuals (full and half-siblings) can bias some genetic diversity and structure analyses, all but one individual from each sibling group were excluded from these tests.

### Genetic diversity and kinship analysis

Average observed (*H*_*o*_) and expected (*H*_*e*_) heterozygosities, allelic richness, private allelic richness, percentage of polymorphic loci and Fixation Index (*FIS*) were calculated with Genalex v6.5 [49]. Effective population size was calculated using the Linkage Disequilibrium method in N_E_ Estimator v2 [50]. Fine scale relatedness was determined using two methods. First, we employed SPAGeDi [51] to calculate Loiselle’s kinship coefficient between pairs of individuals and estimate specific relationships for each pair. We considered kinship coefficients over 0.1875 for full-sibs and between 0.0938 and 0.1875 for half-sibs and negative coefficients for unrelated individuals. Secondly, we also used ML-relate [52] to determine Maximum Likelihood relationships between individuals and establish statistical significance for imputed relationships using the Likelihood Ratio Test with 10000 simulations. For these analyses, samples from Area 1 were analyzed for each season separately whereas specimens from distant sampling sites (LUG and MG) were excluded.

### Spatial genetic structure and temporal stability

To investigate spatial population genetic structure we first performed a hierarchical Analysis of Molecular Variance (AMOVA) for all areas using Genalex with 9999 permutations. Pairwise genetic distances (F_ST_) between areas and between sampling sites were calculated using the R package ade4 with 10000 permutations. Temporal stability of spatial genetic structure was only assessed for the 2017 and 2018 samples obtained in Area 1, with sampling sites nested within seasons.

Spatial genetic structure was evaluated for samples collected in Buenos Aires and for each area separately, using two methods. First, we employed Discriminant Analysis of Principal Components (DAPC) as implemented in the R package Adegenet [53]. The number of clusters was determined using the k-means function *find*.*clusters* and the value of K minimizing Bayesian Information Criterion was selected (S1 Appendix). DAPC function was run retaining the number of principal components previously calculated with a cross-validation analysis to avoid overfitting. Finally, spatial genetic structure was also investigated using the Bayesian clustering method as implemented in STRUCTURE v2.3.4 [54]. We used an admixture model with correlated allele frequencies among populations and previous information on sampling points. The number of burn-ins was 50.000 followed by 500.000 MCMC steps. Estimations were performed from K = 1 to K = N, where N represents the number of sampling sites for each area, and 10 iterations for each value. The most likely number of clusters was estimated using the deltak method proposed by Evanno [55] implemented in Structure Harvester [56]. When the Evanno algorithm yielded K = 2 as the optimal value, we employed Structure Selector [57] in order to examine the possibility that other values of K were in fact the optimal number of clusters (S1 Appendix). STRUCTURE results were processed using Clumpak [58] and plotted with DISTRUCT [59]. Prior to these analyses, we excluded all but one individual from each full-sibling group to avoid including inbred individuals.

Isolation-by-distance was assessed for all samples from Buenos Aires (after pruning the dataset of closely related individuals) by means of Mantel correlation analyses between matrices of genetic and geographic distances with 9999 permutations followed by the Monte-Carlo test, as implemented in Adegenet. In Area 3, IBD analyses were performed for males and females separately to determine whether IBD patterns were sex-specific. We could not perform separate analyses for the other areas because sex distribution in the samples was highly uneven. Spatial autocorrelation analyses were performed using Genalex with 9999 permutations for Area 2 and Area 3 using 100 m intervals to determine the distance at which mosquito specimens were more related. This analysis was not conducted for Area 1 samples because sampling points in this area were more spaced than in the other two.

### Countrywide genetic structure

To determine countrywide genetic structure and to infer the evolutionary history of *Ae*. *aegypti* colonization in Argentina, we conducted a DAPC analysis including the dataset from Buenos Aires (N = 99), samples collected from two sites in Northeastern Argentina (Colonia Aurora -CA- and Eldorado -ED-, N = 30) and a previously published Rad-seq dataset from six locations in Northwestern Argentina (Orán -OR-, Tartagal -TA-, Benjamín Paz -BP-, Tafí Viejo -TV-, Río Hondo -RH- and La Banda -LB-, N = 59) [12]. Pairwise F_ST_ values between populations were calculated with hierfstat package and used to construct a Neighbor-joining unrooted tree as implemented in R package ape [60].

## Results

Mean observed heterozygosity was significantly lower than expected in all areas (Table 1), as also suggested by FIS estimates. The percentage of polymorphic SNPs was higher in Area2, followed by Area 1 (S2017) and Area 3, respectively. LUG and MG presented lower values, although in these cases low sample sizes may affect the outcome of the analysis.

**Table 1:** Basic statistics of population genetic diversity for *Ae*. *aegypti* in different areas of Buenos Aires city.

Location	N	% polymorphic SNPs	N_a_	N_e_	*H*_*o*_	*H*_*e*_	*μHe*	*FIS*
Area 1 (S2017) LUG MG Area 2 Area 3	18 6 5 35 19	92.70 61.77 66.77 99.05 90.38	1904 1617 1666 1990 1904	1481 1385 1417 1503 1476	0.209 0.212 0.220 0.211 0.209	0.288 0.225 0.244 0.303 0.284	0.298 0.250 0.277 0.308 0.293	0.197 -0.075^#^ -0.043^#^ 0.289 0.245

N: sample size, % pol SNPs: percentage of polymorphic SNPs, N_a_: number of alleles, N_e_: number of effective alleles, *H*_*e*_: expected heterozygosity, *H*_*o*_: observed heterozygosity, *μH*_*e*_: unbiased expected heterozygosity, *FIS* inbreeding/fixation index. *FIS* estimates were calculated after excluding all but one specimen from highly related groups.

^#^ Less than 5 samples included for calculations

**Fig 2:**
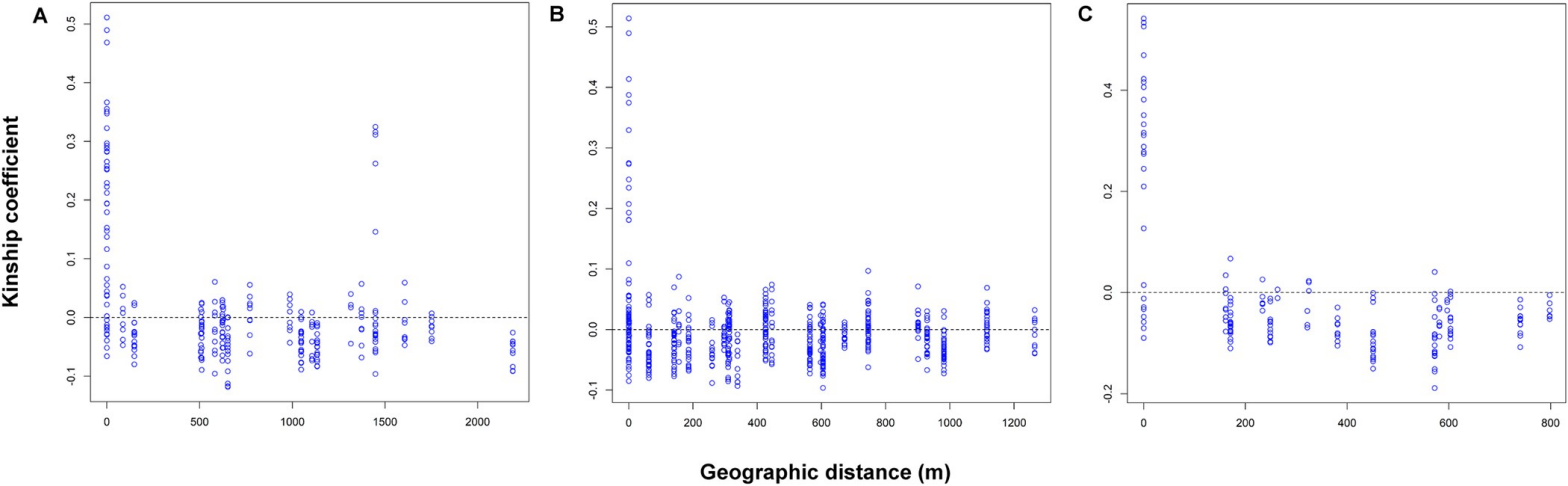
**Loiselle’s kinship coefficients across geographic distances (m) for individual pairs in Area 1 (A), Area 2 (B) and Area 3 (C).** For Area 1, individuals were previously separated according to the collection season.

Kinship analyses using Loiselle’s coefficients and maximum likelihood relatedness yielded similar results. In Area 1, 27 pairs of full siblings were found in the same sites (Fig 2A), 14 pairs in S2017 and 13 pairs in S2018. Interestingly, five pairs of full-sibs located almost 1500 m apart were also detected among the S2017 specimens (Fig 2A). These pairs comprised all samples from site 3 and one sample from site 2 and were grouped into a separated cluster in population structure analyses for this area (Fig 2A). In Area 2, ten pairs of full-siblings and 5 pairs of half-siblings were found in the same sites (Fig 2B) whereas in Area 3, eighteen pairs of full-siblings and two pairs of half-siblings were detected in the same sites (Fig 2C). Loiselle’s kinship coefficients and Maximum likelihood relatedness were significantly correlated (Pearson, p<0.0001) and all relationships were statistically validated with the likelihood ratio test (p<001).

The AMOVA revealed significant spatial population structure among areas (accounting for 5% of total variation, p<0.001). When the analysis was performed for each area separately, we found significant spatial structure that was temporally stable between seasons (p = 0.480) in Area 1. In this area, 13% of total variation was explained by differences among sites within seasons, whereas no variation was detected across seasons. The AMOVA also evidenced significant spatial population structure in Area 2 and Area 3 where variation among sampling sites accounted for 7% (p<0.001) and 30% (p<0.001) of total variation, respectively (Table 2). Pairwise F_ST_ comparisons between areas showed that mosquitoes from LUG displayed highest differentiation with respect to all other locations (Table 3).

**Table 2:** Analysis of Molecular Variance (AMOVA) of *Ae*. *aegypti* samples from Buenos Aires and from each area within the city.

AMOVA summary					
Source of variation	Df	SS	MS	Est. Var.	%
**ALL AREAS**					
**Among areas** **Among individuals within areas** **Error** **Total**	4 77 82 163	8687.79 88972.15 21967.00 119626.93	2171.95 1155.48 267.890	34.80 443.796 267.89 746.49	5* 59* 36* 100
**AREA 1**					
**Among seasons** **Among sampling sites** **Among individuals within sites** **Error** **Total**	1 8 25 35 69	2212.63 15087.57 23740.41 9449.00 50489.60	2212.63 1885.95 949.62 269.97	4.88 138.58 339.82 269.97 753.26	1 18* 45* 36* 100
**AREA 2**					
**Among sites** **Among individuals within sites** **Error** **Total**	4 13 18 35	9059.40 9664.79 4868.50 23592.69	2264.85 743.45 270.47	219.08 236.49 270.47 726.04	30* 33* 37* 100
**AREA 3**					
**Among sites** **Among individuals within sites** **Error** **Total**	6 28 35 69	9536.67 31317.04 9415.00 50268.71	1589.45 1118.47 269.00	48.29 424.73 269.00 742.03	7* 57* 36* 100

For Area 1, AMOVA was performed with sampling sites nested within season. The * symbol marks significant differences.

**Table 3:** Pairwise F_ST_ values for *Ae*. *aegypti* collected in Buenos Aires.

Sampling site	Area 1	LUG	MG	Area 2	Area 3
**Area 1 (S2017)**		0.100	0.047	0.023	0.050
**LUG**	0.100		0.120	0.080	0.115
**MG**	0.047	0.120		0.034	0.064
**Area 2**	0.023	0.080	0.034		0.033
**Area 3**	0.05	0.115	0.064	0.033	

All F_ST_ values were significant according to probability P (rand > = data) based on 9999 permutations (p<0.05).LUG = Lugano, MG = Monte Grande.

**Fig 3:**
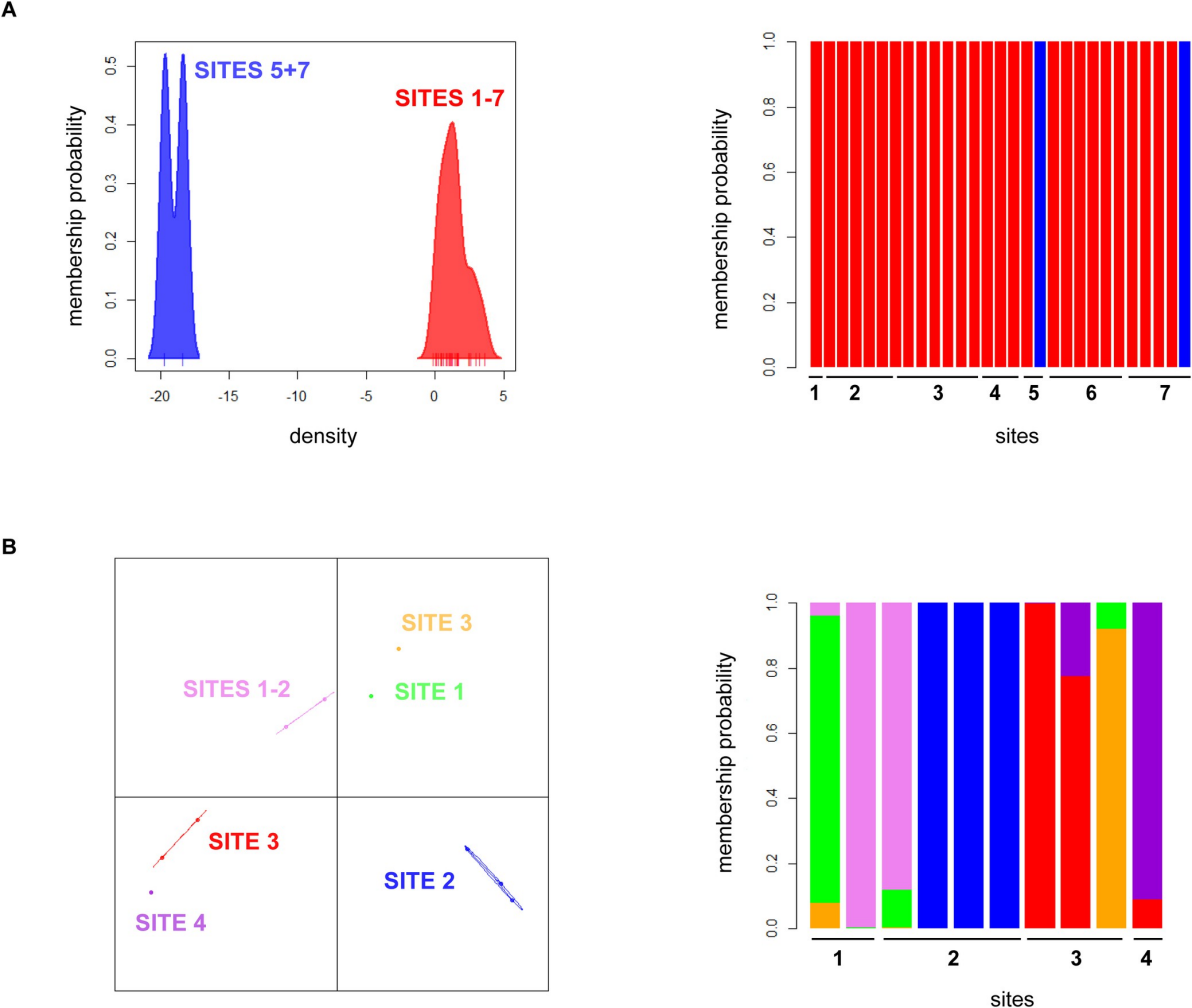
**Comparative DAPC analysis for *Ae*. *aegypti* samples from Area 2 (A) and Area 3 (B) in Buenos Aires city.** On the left, scatter plot with individuals (curve area/dots) assigned to the detected clusters using the k means function find.clusters. On the right, individual membership probability to each cluster (vertical lines) using compoplot in Adegenet. Clustering analyses for samples from Area 1 yielded only one cluster and therefore were not included in DAPC analyses.

**Fig 4:**
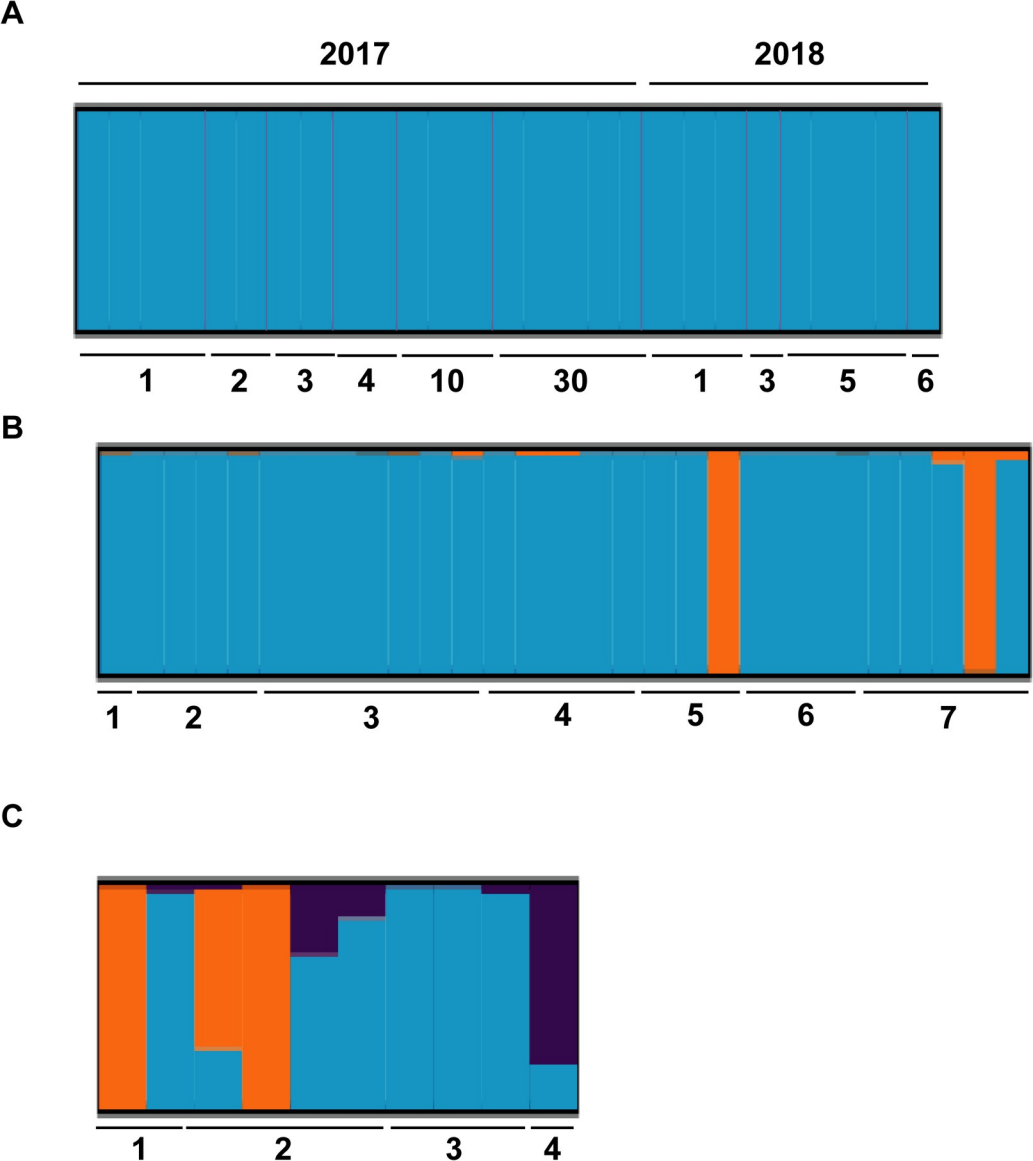
**STRUCTURE analysis for *Ae*. *aegypti* samples from Area 1 (A), Area 2 (B) and Area 3 (C) in Buenos Aires City.** Individual membership probability to each cluster is depicted with vertical lines with different colours representing each cluster.

The analysis of spatial genetic structure using DAPC considering all samples from Buenos Aires yielded K = 1 as the optimal value, as indicated by the lowest value of BIC used in the find.clusters function. When DAPC was applied to analyze differentiation among sites within each area, we found different patrons of spatial structure. Samples from Area 1 comprised one genetic cluster, evidencing no sign of genetic structure in this area. *Ae*. *aegypti* individuals collected in Area 2 appeared distributed into 2 separate clusters: one comprising 2 specimens from sites 5 and 7, respectively and another one including all remaining samples (Fig 3A). For Area 3, samples from each site presented a high degree of inbreeding, even those obtained from different ovitraps within the same site or sampled one week apart from the same sensor. In order to perform DAPC and STRUCTURE analyses for this area, we excluded all but one individual from full-siblings groups collected in the same ovitrap except for a pair of full-siblings from site 4 that were recovered in successive weeks. We also kept a pair of half-siblings from this same site. Because sample sizes were smaller in this area, we grouped individuals from nearby sites in 4 sampling sites for these analyses. DAPC for this area yielded 6 clusters, mostly corresponding to different sampling sites (Fig 3B),

Analysis with STRUCTURE using the deltaK method for all samples from Buenos Aires detected four clusters, though they presented admixture and included samples from different locations (cluster 1 = three samples from MG, cluster 2 = two samples Area 1 Site 3 (2017) + one sample from Area 1 Site2, cluster 3 = one sample from Area 1 Site 3 (2018) + one sample from Area 1 Site 1, cluster 4 = all remaining samples) (S1 Fig). Further analyses per area detected two clusters (K = 2) after removing highly related individuals in Area 1.However, Structure selector algorithm yielded similar results as Adegenet (K = 1) (Fig 4A). Migrant detection using Geneclass2 for specimens in Area 1 showed that two individuals from MG (located 30 km away) were first generation migrants (p<0.01) originated from site 2 in the city. In Area 2, STRUCTURE found two clusters (K = 2) both by Evanno and Structure selector algorithms, one including a sample from site 5 and another one from site 7 (Fig 4B).STRUCTURE also revealed genetic structure in Area 3. Two clusters were detected using Evanno method, with two specimens from site 2 separated from the rest. When we used Structure selector, 3 clusters were detected. In this case, the same cluster that included two out of four samples from site 2, a cluster including the unique sample from site 4 and another one encompassing the remaining samples (Fig 4C). However, these results indicative of moderate structure should be taken with caution because of the low sample size obtained after exclusion of all but one individual from each full-sibling group.

Isolation-by-distance was not significant for *Ae*. *aegypti* from all three areas of Buenos Aires city (r = 0.04, p = 0.304) (S2 Fig). When Mantel tests was performed within each area, only samples from Area 2 showed a significant IBD pattern (Mantel, r = 0.209, p = 0.046). When the analysis was performed separately for each sex, we found a slightly more significant pattern in females than males (r = 0.292 p = 0.01 for females and r = 0.211 p = 0.017 for males) (S3 Fig). Fine-scale autocorrelation analysis performed for Areas 2 and 3 showed that higher relatedness values for pairs of individuals could be found at distances below 100 meters in both areas (S4 Fig). Estimates of effective population size were higher in Area 2 with respect to Area 1 although 95% confidence intervals for Area 1 and Area 2 partially overlapped (Table 4).

**Table 4:** Estimation of effective population size for all areas using linkage disequilibrium method.

Location	Linkage disequilibrium Ne Estimate	Approx. 95% Confidence Interval
**Area 1 (S2017)** **Area 2**	56.6 210.5	28.7–171.5 79.5 –Infinite

^**#**^ **Samples from Area 3 were not included in this analysis due to low sample size and high inbreeding.**

**Fig 5:**
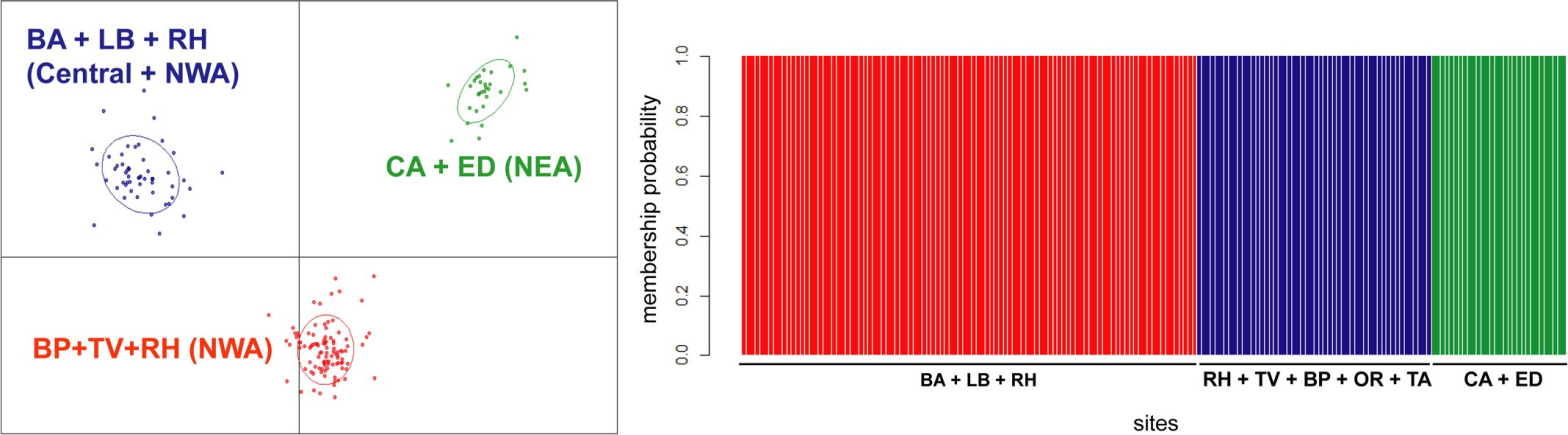
DAPC analysis for *Ae*. *aegypti* samples from different locations in Northwestern, Northeastern and Central Argentina. On the left, scatter plot with individuals (dots) assigned to the three detected clusters using the k means function find.clusters. On the right, individual membership probability to each cluster (vertical lines).

**Fig 6:**
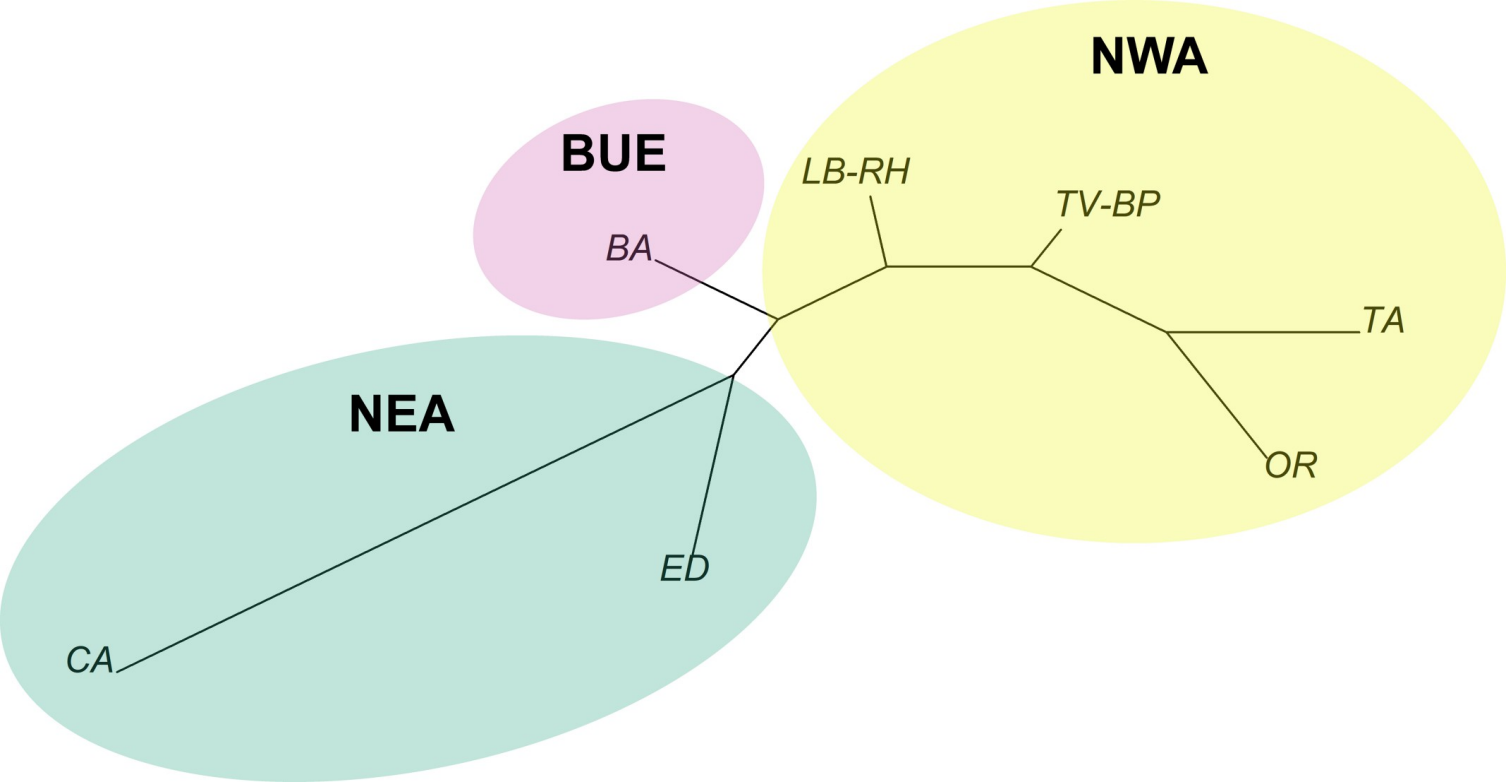
Unrooted Neighbor-joining tree for *Ae*. *aegypti* specimens collected in different regions of Argentina. The tree was constructed using a genetic distances matrix (Nei’s F_ST_ as implemented in R package ape).

Country-wide genetic structure of *Ae*. *aegypti* in Argentina performed with DAPC in Adegenet showed that *Ae*. *aegypti* populations were clearly differentiated at a regional level (Fig 5). This analysis revealed three clusters: one encompassing samples from the two localities of NEA (CA and ED); the second including specimens from Buenos Aires and 4 individuals from the nearest NWA localities (LB and RH) and the third cluster encompassing the remaining samples from NWA (OR, TA, RH, TV, BP) (Fig 5). These patterns are coincident with F_ST_ values (Table 5) and the relationships depicted in the Neighbor-joining tree based on genetic distances among populations (Fig 6). In effect, samples collected in Buenos Aires occupy an intermediate position between NEA and NWA, the NWA sites LB and RH appear closer to Buenos Aires and CA appears as the most divergent population.

**Table 5:** Pairwise F_ST_ values for *Ae*. *aegypti* collected in Buenos Aires and in Northeastern and Northwestern Argentina localities.

Location	CA	ED	BA	LB-RH	TV-BP	OR	TA
**CA**		0.124	0.127	0.134	0.152	0.194	0.196
**ED**	0.124		0.057	0.066	0.084	0.117	0.116
**BA**	0.127	0.057		0.047	0.064	0.102	0.104
**LB-RH**	0.134	0.066	0.047		0.036	0.075	0.084
**TV-BP**	0.152	0.084	0.064	0.036		0.047	0.060
**OR**	0.194	0.117	0.102	0.075	0.047		0.050
**TA**	0.196	0.116	0.104	0.084	0.060	0.050	

All F_ST_ values were significant according to probability P(rand > = data) based on 9999 permutations (p<0.05). CA = Colonia Aurora, ED = Eldorado, BA = Buenos Aires, LB-RH = La Banda-Río Hondo, TV-BP = Tafí Viejo-Benjamín Paz, OR = Orán, TA = Tartagal.

## Discussion

Our study indicates that spatial genetic structure and population dynamics in *Ae*. *aegypti* are conditioned by urbanization levels and resource availability (breeding sites, human hosts and nectar). Populations living in less urbanized city areas with more vegetation cover tend to be more structured than those from highly urbanized areas. Clustering analysis at the city level evidenced the presence of weak genetic structure in *Ae*. *aegypti* from Buenos Aires. These results are in accordance with previous studies reporting nil/low spatial structure in other cities and regions [17,22,23]. It is interesting to point out that, in the case of Buenos Aires, this genetic pattern was achieved in a relatively short period of time (~27 years) when compared with other locations, and in a temperate climate that determines the interruption of the vector’s life cycle during the cold season.

Further genetic analysis at a smaller scale allowed us to detect subtle spatial patterns in areas with variable degrees of urbanization. Our results revealed an absence of genetic structure in Area 1. Interestingly, specimens collected in the city’s outskirts (30 km apart) clustered together with samples from dowtown Buenos Aires, suggesting a role of human-mediated passive transport in shaping population structure and promoting gene flow between distant populations. In addition, our results show that urban barriers such as two-ways streets do not seem to restrict gene flow in highly urbanized Area 1.

Buenos Aires city is located in a temperate area where *Ae*. *aegypti* populations usually pass the winter season in the egg stage [61] with adults starting to emerge around late September. Genetic structure did not appear to be associated with seasonal variation at least in the single comparison between two successive mosquito generations. Our results on this matter were coincident with previous reports in subtropical or other temperate areas where genetic structure was reported to be stable between wet and dry seasons [16,23,62,63]. However, these results should be interpreted with caution since more extensive temporal sampling sin several points are necessary to confirm if such stability is a generalized feature within the city. The lack of significant variation between samples collected at the end of the season (early autumn) and at the beginning of the next season (early spring) suggests that no effective control measures were taken against *Ae*. *aegypti* during the winter, at least within a reduced urban environment. Although it is the main metropolis in Argentina and has had a stable population of *Ae*. *aegypti* for the last 27 years, Buenos Aires does not have sustainable control programs against the dengue vector. Winter arrest could provide a valuable opportunity to tackle breeding sites when no adults are emerging. The worryingly increase in dengue prevalence during the last epidemics (2016, 2020), suggests that immediate actions against the vector are needed.

Our analyses yielded two clusters in Area 2 where the main group included the vast majority of specimens from all sampling sites in this area. Interestingly, samples collected in the neighbourhood adjacent to Chacarita cemetery clustered together with samples from the cemeteries. The neighbourhood adjacent to the cemeteries presented high prevalence of dengue fever during the 2016 epidemic, highlighting the potential role of cemeteries as vector reservoirs and source of arboviral transmission. Overall, there are few features that may serve as barriers for mosquito flight in cemeteries since buildings are separated and internal streets are rather narrow and present low circulation. Our results also suggest that the wall separating both cemeteries functions as a partial barrier for *Ae*. *aegypti* active dispersal, given that the separated clusters were detected on both sides of the wall.

Finally, Area 3 showed the highest number of clusters. However, this result should be interpreted with caution due to the low sampling size resulting after the removal of closely related individuals. In most cases, family clusters were associated with a unique collection site, pointing to a close dependence between mosquitoes and human dwellings within the area. Area 3 presents a high percentage of vegetation coverage and buildings arranged in isolated patches, which creates a discontinuous distribution of potential breeding sites and human host availability for *Ae*. *aegypti*. Passive transport of immature stages between sites does not seem to play an important role in shaping population structure in this area, probably because permanent human presence is mostly restricted to sites 1 and 6. Elevated inbreeding in this area hindered the possibility of obtaining samples from non-related individuals. Although we placed several ovitraps between building patches, we were not able to obtain eggs from these locations. Siblings were recovered not only from the same ovitraps but also from separated containers within the same site or even in ovitraps placed in the same place in successive weeks. Even though in this case genetic clusters are conditioned by low sampling sizes, we believe that our approach allowed us to unveil the role of urban landscapes in shaping population dynamics in *Ae*. *aegypti*. It also points out the need to perform kinship analysis prior to genetic analysis, even when other preventive measures to avoid oversampling of highly related individuals were *a priori* considered (eg. selecting a low number of specimens from each ovitrap/container).

Even though Area 3 is isolated from the rest of the city, circulation of people is restricted to daytime hours and permanent human residents are scarce, *Ae*. *aegypti* has been present in this area for at least the last 27 years. Active dispersal from other parts of the city to this area is highly unlikely since Ciudad Universitaria is surrounded by the Río de la Plata to the east and by two highways with heavy traffic that connect the north and southern bounds of the city to the west. Previous studies in other cities have shown that highways can constitute effective barriers for *Ae*. *aegypti* active dispersal [24]. However, passive transport of adults or containers with eggs and/or immature stages represent the most probable way of introduction of mosquitoes into this area. In fact, during the previous months of this study, different construction sites begun to operate in the premises. These sites may have represented an introduction source of mosquitoes carrying novel genetic variants and, probably, contributed to maintain the existing population. Indeed, there is evidence that construction sites represent favourable habitats for mosquitoes adapted to live in human altered environments such as *Ae*. *aegypti* or *Culex quinquefasciatus* [64].

Isolation-by-distance was not significant in Buenos Aires, though mosquitoes from Area 2 displayed a significant IBD pattern, confirming the crucial role of active dispersal in shaping genetic structure in this poorly urbanized area. Previous reports have shown that size and density of oviposition sites influence the dispersal of *Ae*. *aegypti* [65,66]; in fact, availability of containers was shown to be inversely correlated with female dispersal ability. However, this relationship may be mediated by environmental and microclimate features [66]. In Area 3, buildings and therefore artificial containers are scarce and patchily distributed, a fact that may prevent female dispersal. Differences in vegetation cover and land use can account for the different IBD patterns between Areas 1 and 2. In the highly urbanized Area 1, we did not detect a significant IBD pattern despite the likely continuous availability of potential breeding sites. Previous studies reported an increase in the number of potential oviposition sites for the vector in recent years in several neighbourhoods of Buenos Aires city [34,67,68] in the context of an ongoing colonization process in the area [69]. In addition, IBD analyses performed separately for each sex in Area 2 showed that males disperse more than females, confirming previous findings in catch-release experiments [70]. Accurate estimation of female dispersal remains a key feature for vector control programs, particularly those involving oviposition sites removal and focal insecticide spraying.

Kinship analysis detected full and half siblings in the same ovitraps in all areas. Also, a pair of full-siblings was found in Area 1 in sites located over 1500 meters apart, which sets an upper bound of dispersal ability, though passive transport cannot be ruled out. Effective population size was higher in Area 2than in Area 1. The higher Ne value could be caused by the exceptional conditions that cemeteries offer *Ae*. *aegypti*, with a continuous distribution of flower containers next to graves that provide suitable oviposition sites, as has been shown in previous studies in the same areas [26,41].

Our results provide relevant information for the improvement of current control measures and also for the implementation of novel control strategies such as the release of *Wolbachia*- infected mosquitoes. The absence of extensive genetic clustering of populations from urbanized areas could favour *Wolbachia* invasion. At the same time, less urbanized areas within the city with more structured populations might require an increased rate of releases to favour the spread of *Wolbachia*-infected mosquitoes.

The countrywide survey of *Ae*. *aegypti* showed clear regional clustering. Samples from Buenos Aires, located in the southern limit of the vector’s distribution, were differentiated from samples collected in NEA and NWA. It is interesting to point out that some specimens collected in intermediate NWA localities clustered with samples from Buenos Aires, suggesting that passive transport from the Northwest along the Panamerican route that connects Buenos Aires and NWA, is an important source of novel genetic variants into the city. Likewise, the neighbour-joining analysis based on genetic distances (F_ST_) also showed that samples from Buenos Aires are closely related to samples from these intermediate Northwestern locations but also with samples collected in the Northeastern locality of Eldorado (ED). Previous studies suggested that *Ae*. *aegypti* from Buenos Aires might be more closely related to Northeastern populations [42]. Our results confirm this hypothesis, but also provide evidence that southern populations in Buenos Aires are more connected to the Northwest than previously assumed. Buenos Aires city represents the centre of Argentina’s productive matrix, all main land routes originate or pass through Buenos Aires since it concentrates most of the country’s commercial and productive activity. Therefore, intense traffic from and to NWA and NEA occurs on a daily basis and creates ideal conditions for a constant exchange of eggs and immature stages. This could place *Ae*. *aegypti* populations from Buenos Aires as a source of novel genetic variants originated from the admixture of NEA, NWA and local vector populations.

In this work, *we* provide a first insight into spatial genetic structure of *Ae*. *aegypti* in temperate Buenos Aires. Our results show that both urbanization levels as well as the distribution of potential breeding sites and meal sources represent crucial features that condition mosquito dispersal and determine population genetic structure. Vector control programs should consider urban landscape heterogeneity in order to develop comprehensive and successful prevention strategies against *Ae*. *aegypti*.

## Supporting information

S1 Fig STRUCTURE analysis for *Ae*. *aegypti* samples from Buenos Aires city. Individual membership probability to each cluster is depicted with vertical lines with different colours representing each cluster.
(TIF)

S2 Fig Isolation-by-distance plots for *Ae*. *aegypti* specimens from Buenos Aires. On the left, the dot represents the original value of the correlation between the matrices of Edwards’ genetic distances and Euclidean geographic distances whereas the histogram depicts permuted values (with 9999 replicates). On the right, the graph shows the relationship between genetic and geographic distances.
(TIF)

S3 Fig **Isolation-by-distance plots for *Ae*. *aegypti* male (A) and female (B) specimens from Area 2.** On the left, the dot represents the original value of the correlation between the matrices of Edwards’ genetic distances and Euclidean geographic distances whereas the histogram depicts permuted values (with 9999 replicates). On the right, the graph shows the relationship between genetic and geographic distances for females (r = 0.164, p = 0.016) and males (r = 0.292, p = 0.004).
(TIF)

S4 Fig **Spatial autocorrelation analysis for Ae. aegypti specimens from Area 2 (A) and Area 3 (B) separated ≤800 m apart.** Upper and lower confidence interval for autocorrelation coefficient (r) were calculated using 9999 permutations. Distance classes for which significant positive spatial autocorrelation [p(r-rand≥ r-data)< 0.05] are marked with the * symbol.
(TIF)

S1 Appendix STRUCTURE (DeltaK algorithm and Structure Selector results) and Adegenet (BIC vs. Cluster numbers) analyses used to perform cluster assignation. (DOCX)

S1 Table Spatial coordinates for all samples from Buenos Aires used in this study. (TXT)
