# Supplementary figures and images for: City puzzles: Does urban land scape affect genetic population structure in Aedes aegypti?
Source: PLoS Negl Trop Dis. 2022 Jul 6;16(7):e0010549. doi: 10.1371/journal.pntd.0010549 (PMC9292108; doi:10.1371/journal.pntd.0010549)

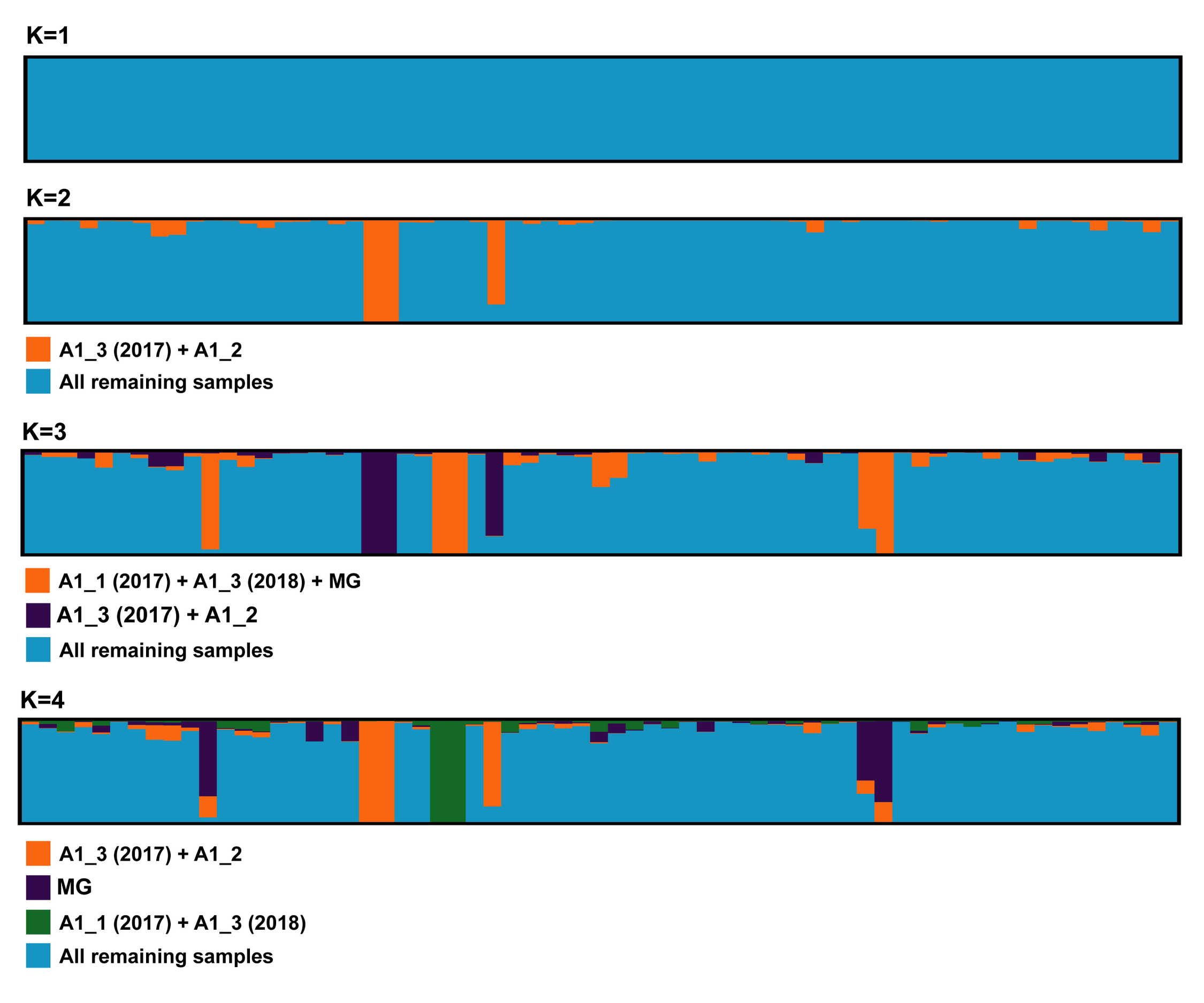

Supplement: S1 Fig — Individual membership probability to each cluster is depicted with vertical lines with different colours representing each cluster. (TIF) [file pntd.0010549.s001.tif]

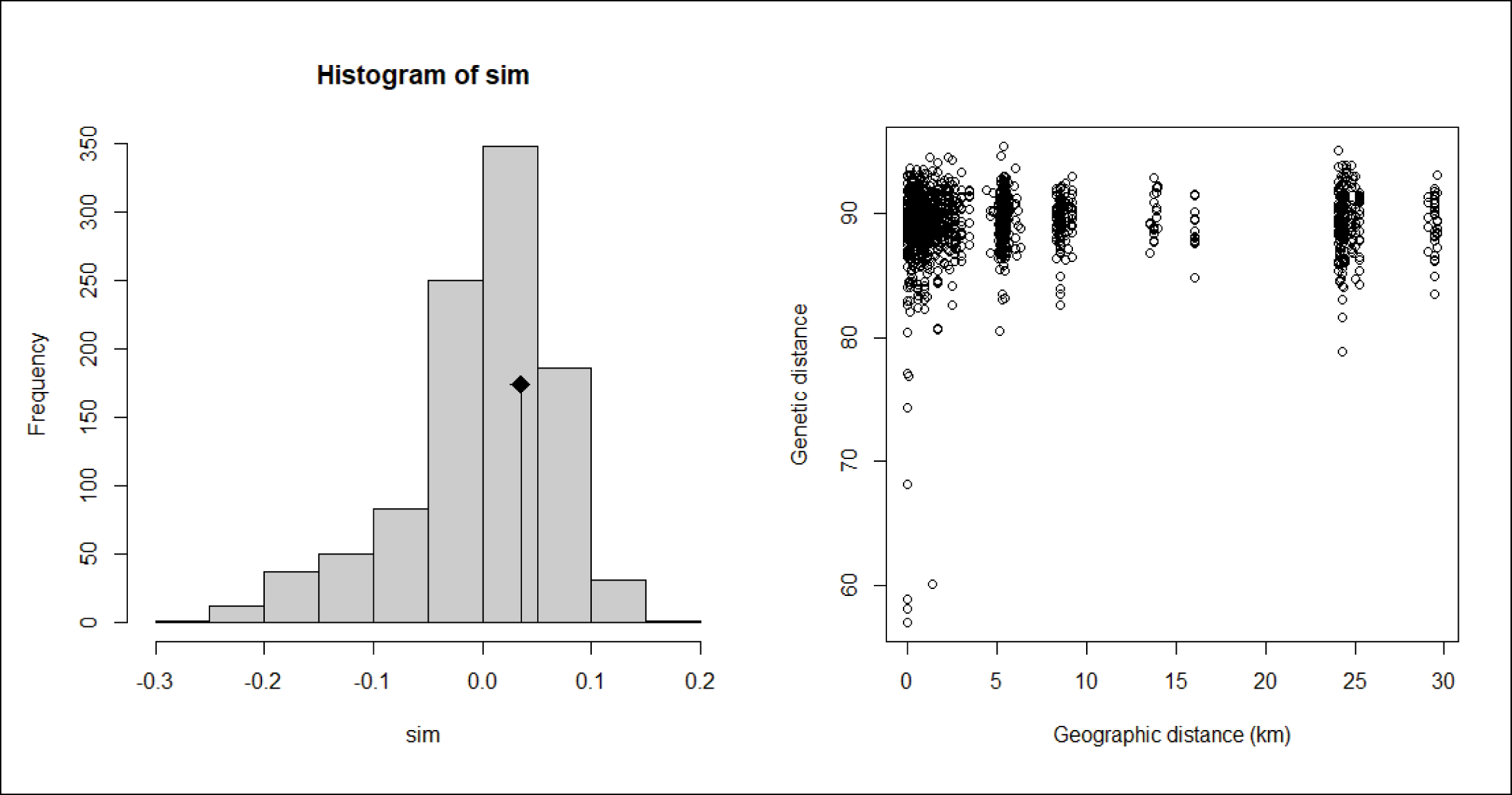

Supplement: S2 Fig — On the left, the dot represents the original value of the correlation between the matrices of Edwards’ genetic distances and Euclidean geographic distances whereas the histogram depicts permuted values (with 9999 replicates). On the right, the graph shows the relationship between genetic and geographic distances. (TIF) [file pntd.0010549.s002.tif]

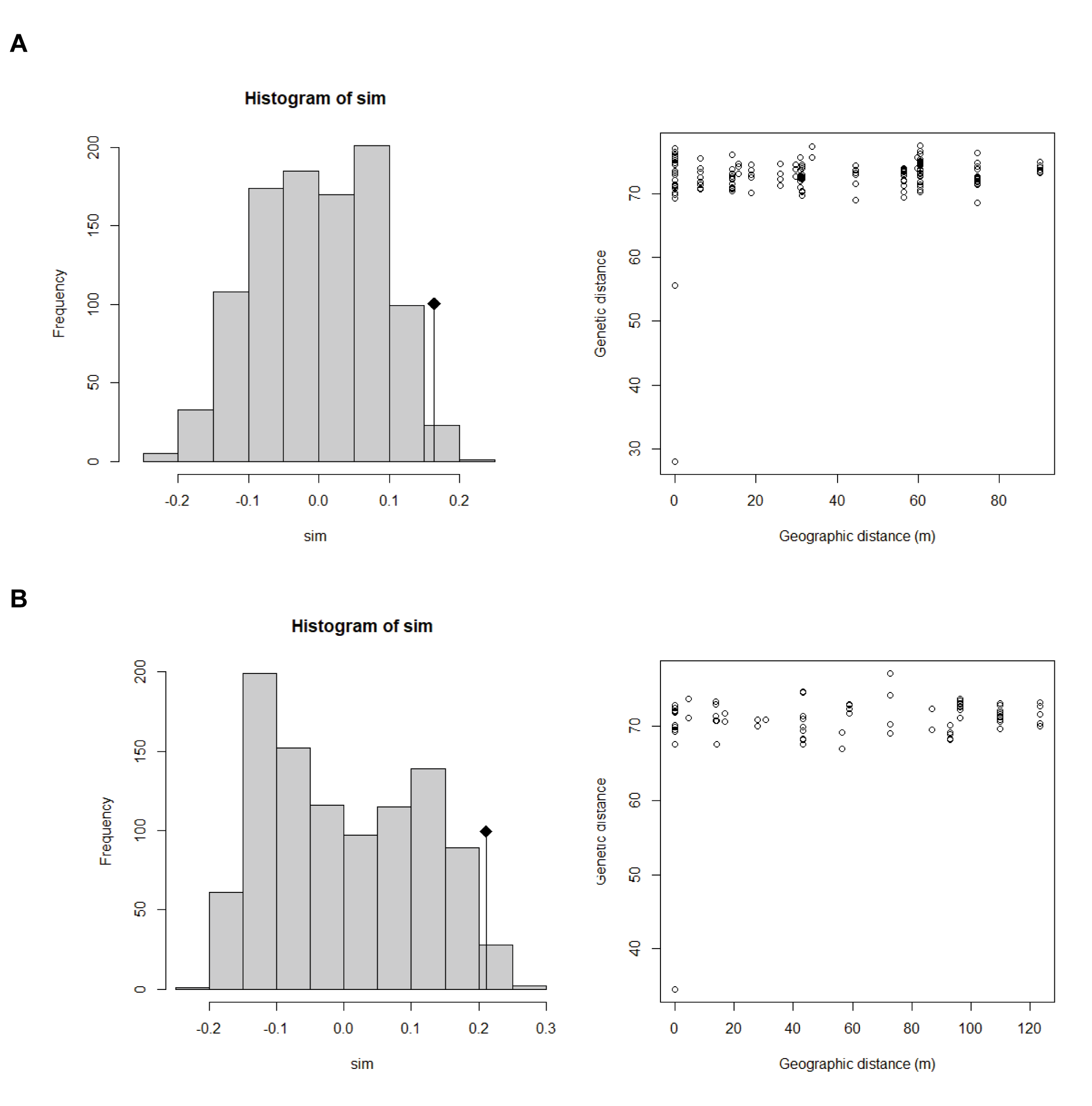

Supplement: S3 Fig — Isolation-by-distance plots for Ae. aegypti male (A) and female (B) specimens from Area 2. On the left, the dot represents the original value of the correlation between the matrices of Edwards’ genetic distances and Euclidean geographic distances whereas the histogram depicts permuted values (with 9999 replicates). On the right, the graph shows the relationship between genetic and geographic distances for females (r = 0.164, p = 0.016) and males (r = 0.292, p = 0.004). (TIF) [file pntd.0010549.s003.tif]

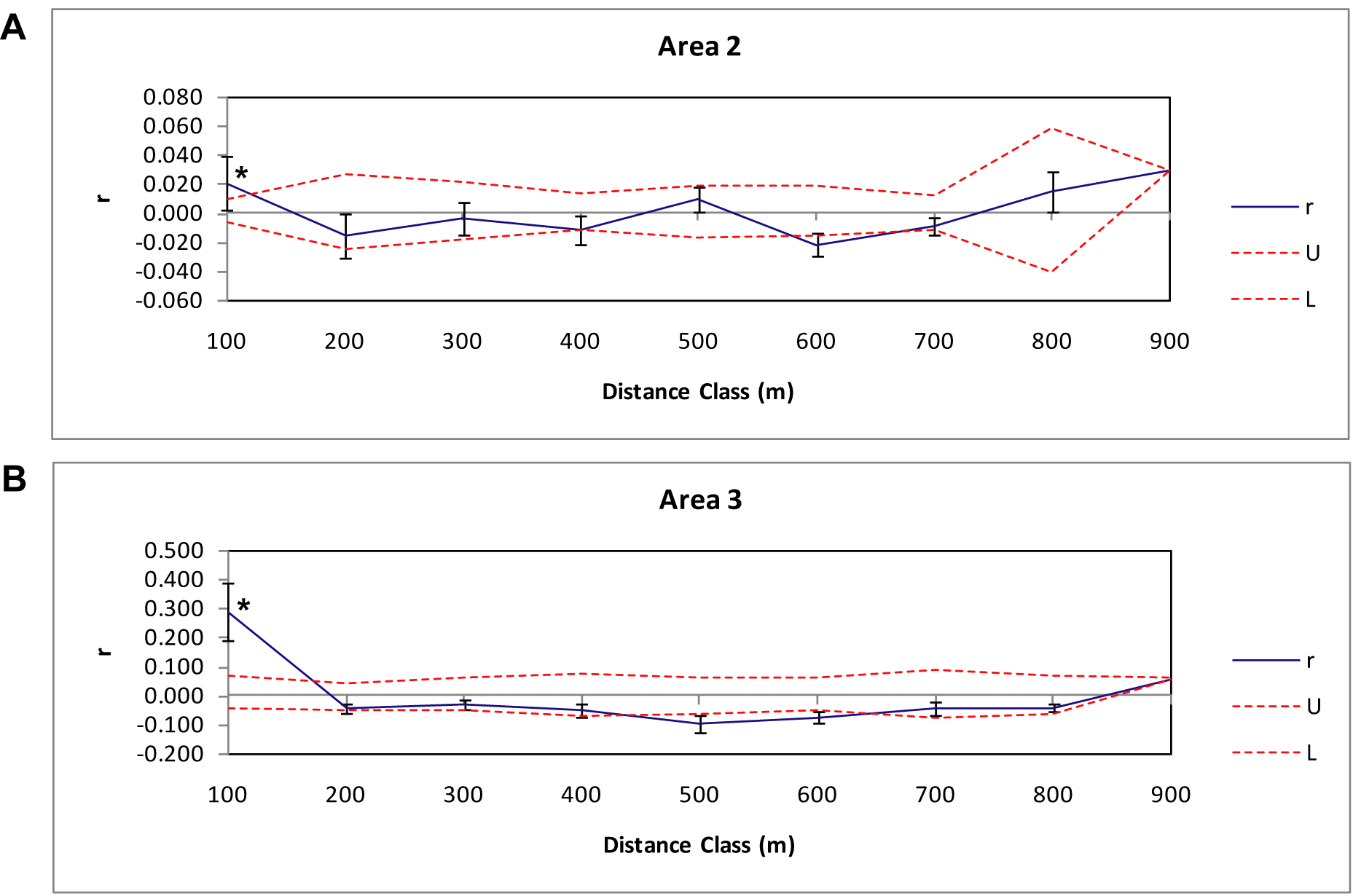

Supplement: S4 Fig — Spatial autocorrelation analysis for Ae. aegypti specimens from Area 2 (A) and Area 3 (B) separated ≤800 m apart. Upper and lower confidence interval for autocorrelation coefficient (r) were calculated using 9999 permutations. Distance classes for which significant positive spatial autocorrelation [p(r-rand≥ r-data)< 0.05] are marked with the * symbol. (TIF) [file pntd.0010549.s004.tif]
