# Supplementary material for: City puzzles: Does urban land scape affect genetic population structure in Aedes aegypti?
Source: PLoS Negl Trop Dis. 2022 Jul 6;16(7):e0010549. doi: 10.1371/journal.pntd.0010549 (PMC9292108; doi:10.1371/journal.pntd.0010549)
Supplement: S1 Appendix — (DOCX) [file pntd.0010549.s005.docx]

**Results from Adegenet and STRUCTURE used to perform cluster assignation**

**1. All areas**

**STRUCTURE RESULTS**

# K Reps Mean LnP(K) Stdev LnP(K) Ln'(K) |Ln''(K)| Delta K

1 10 -262263.36000 2.13612 NA NA NA

2 10 -260128.08000 558.81882 2135.28000 169.58000 0.30346

3 10 -258162.38000 14.03841 1965.70000 139.94000 9.96836

**4 10 -256336.62000 57.06235 1825.76000 1104.98000 19.36443**

5 10 -255615.84000 1602.02751 720.78000 1791.93500 1.11854

6 10 -253103.12500 148.51171 2512.71500 1802.46500 12.13685

7 10 -252392.87500 22.76230 710.25000 350.15000 15.38289

8 10 -252032.77500 413.68534 360.10000 335.10000 0.81004

9 10 -251337.57500 122.94376 695.20000 322.97500 2.62701

10 10 -250965.35000 139.21783 372.22500 NA NA

**ADEGENET RESULTS**

**
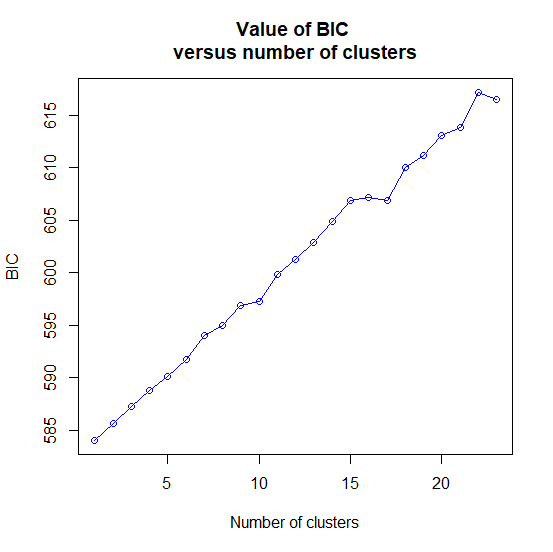
**

**2. Area 1**

**STRUCTURE RESULTS**

**A. Cluster assignation using Evanno et al. method [1]**

# K Reps Mean LnP(K) Stdev LnP(K) Ln'(K) |Ln''(K)| Delta K

1 10 -112159.55000 10.16117 NA NA NA

**2 10 -109862.71000 13.30225 2296.84000 700.03000 52.62493**

3 10 -108265.90000 305.70147 1596.81000 517.25000 1.69201

4 10 -107186.34000 139.68493 1079.56000 1354.48000 9.69668

5 10 -107461.26000 2543.53979 -274.92000 329.30000 0.12947

1. 10 -108065.48000 2234.87892 -604.22000 NA NA

**B. Cluster assignation using Puechmaille method:**

K MedMed MedMean MaxMed MaxMean Reps

1 1 1 1 1 10

2 1 1 1 1 10

3 1 1 1 1 10

4 1 1 1 1 10

5 1 1 1 1 10

6 1 1 1 1 10

MedMedK MedMeaK MaxMedK MaxMeaK

ALL 1 1 1 1

**ADEGENET RESULTS**

**
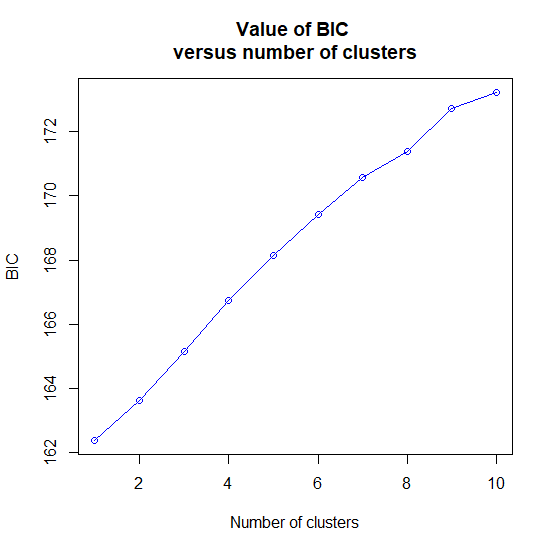
**

**3. Area 2**

**STRUCTURE RESULTS**

**A. Cluster assignation using Evanno et al. method [1]**

# K Reps Mean LnP(K) Stdev LnP(K) Ln'(K) |Ln''(K)| Delta K

1 10 -120146.52000 5.82984 NA NA NA

**2 10 -118406.00000 30.73459 1740.52000 1843.84000 59.99234**

3 10 -118509.32000 91.22534 -103.32000 83.18000 0.91181

4 10 -118529.46000 119.17631 -20.14000 393.50000 3.30183

5 10 -118943.10000 549.69941 -413.64000 501.94000 0.91312

6 10 -118854.80000 257.65589 88.30000 454.30000 1.76320

7 10 -119220.80000 456.20961 -366.00000 NA NA

**B. Cluster assignation using Puechmaille method:**

K MedMed MedMean MaxMed MaxMean Reps

1 1 1 1 1 10

2 1 1 1 1 10

3 2 2 2 2 10

4 2 2 3 3 10

5 2 2 2 3 10

6 1 1 2 3 10

7 1 2 2 2 10

MedMedK MedMeaK MaxMedK MaxMeaK

ALL 2 2 3 3

**ADEGENET RESULTS**

**
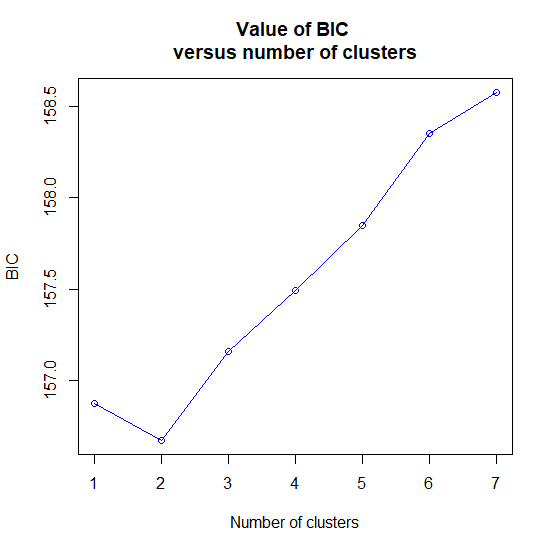
**

**4. Area 3**

**STRUCTURE RESULTS**

**Cluster assignation using Evanno et al. method [1]**

K Reps Mean LnP(K) Stdev LnP(K) Ln'(K) |Ln''(K)| Delta K

1 10 -40702.76000 13.04235 NA NA NA

**2 10 -39028.76000 22.12675 1674.00000 6659.20000 300.95701**

3 10 -44013.96000 3131.50616 -4985.20000 6969.88000 2.22573

4 10 -42029.28000 1916.59137 1984.68000 2035.50000 1.06204

5 10 -42080.10000 2076.44214 -50.82000 495.28000 0.23852

6 10 -42626.20000 2864.28672 -546.10000 NA NA

**K MedMed MedMean MaxMed MaxMean Reps**

**1 1 1 1 1 10**

**2 2 2 2 2 10**

**3 3 3 3 3 10**

**4 3 3 4 4 10**

**5 2 2 3 3 10**

**6 3 3 3 3 10**

**MedMedK MedMeaK MaxMedK MaxMeaK**

**3 3 4 4**

**ADEGENET RESULTS**

**
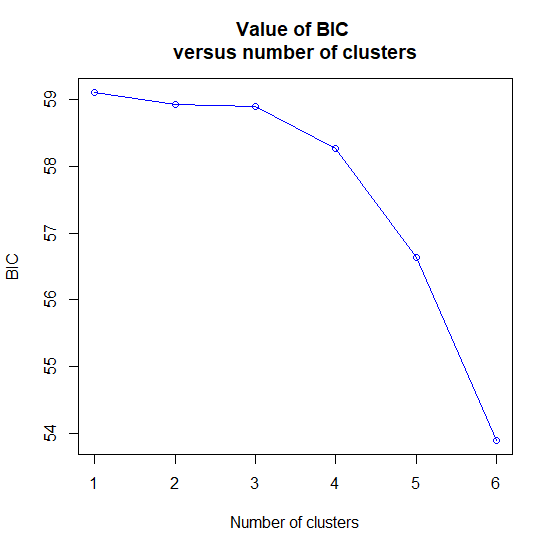
**

**5. Argentina**

**ADEGENET RESULTS**

**
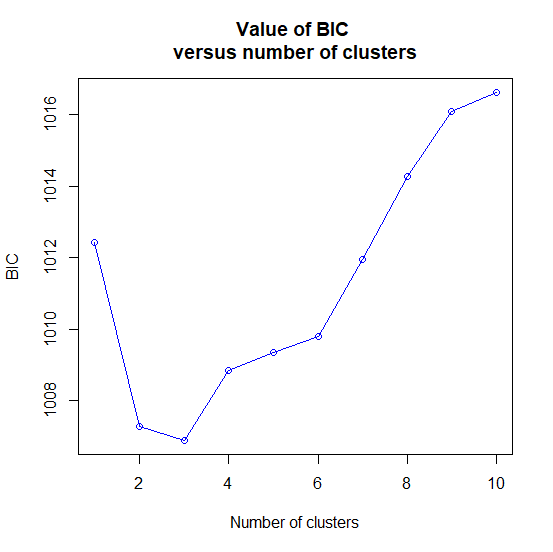
**

References

1. Evanno G, Regnaut S, Goudet J. Detecting the number of clusters of individuals using the software STRUCTURE: a simulation study. Mol Ecol [Internet]. 2005;14(8):2611–20. Available from: http://www.ncbi.nlm.nih.gov/pubmed/15969739
